# Does Social Capital Promote Physical Activity? A Population-Based Study in Japan

**DOI:** 10.1371/journal.pone.0012135

**Published:** 2010-08-12

**Authors:** Kazumune Ueshima, Takeo Fujiwara, Soshi Takao, Etsuji Suzuki, Toshihide Iwase, Hiroyuki Doi, S. V. Subramanian, Ichiro Kawachi

**Affiliations:** 1 Department of Epidemiology, Okayama University Graduate School of Medicine, Dentistry and Pharmaceutical Sciences, Okayama, Japan; 2 Section of Behavioral Science, Department of Health Promotion, National Institute of Public Health, Saitama, Japan; 3 Department of Society, Human Development and Health, Harvard School of Public Health, Boston, Massachusetts, United States of America; Yale University School of Medicine, United States of America

## Abstract

**Background:**

To examine the association between individual-level social capital and physical activity.

**Methodology/Principal Findings:**

In February 2009, data were collected in a population-based cross-sectional survey in Okayama city, Japan. A cluster-sampling approach was used to randomly select 4,000 residents from 20 school districts. A total of 2260 questionnaires were returned (response rate: 57.4%). Individual-level social capital was assessed by an item inquiring about perceived trust of others in the community (cognitive dimension of social capital) categorized as low trust (43.0%), mid trust (38.6%), and high trust (17.3%), as well as participation in voluntary groups (structural dimension of social capital), which further distinguished between bonding (8.9%) and bridging (27.1%) social capital. Using logistic regression, we calculated the odds ratios (ORs) and 95% confidence intervals (CIs) for physical inactivity associated with each domain of social capital. Multiple imputation method was employed for missing data. Among total participants, 68.8% were physically active and 28.9% were inactive. Higher trust was associated with a significantly lower odds of physical inactivity (OR = 0.58, 95% CI = 0.42–0.79) compared with low trust. Both bridging and bonding social capital were marginally significantly associated with lower odds of physical inactivity (bridging, OR = 0.79, 95% CI = 0.62–1.00; bonding, OR = 0.71, 95% CI = 0.48–1.03) compared with lack of structural social capital.

**Conclusions/Significance:**

Low individual-level social capital, especially lower trust of others in the community, was associated with physical inactivity among Japanese adults.

## Introduction

In 1995, the Centers for Disease Control and Prevention and the American College of Sports Medicine recommended a moderate amount of physical activity (e.g., 30 minutes of brisk walking) on most, and preferably all, days of the week for every adult, in order to reduce premature mortality and morbidity [1], [2]. In addition, the US Surgeon General summarized considerable evidence which indicated that physical inactivity is among the strongest predictors of premature mortality [3].

Several factors have been established as individual-level determinants of physical inactivity, including socioeconomic status [4], [5] and educational attainment [6], [7]. In addition, studies have also demonstrated that exercise is linked with barriers as well as enabling factors at the neighborhood level, including features of the built environment (mixed land use, street connectivity), walkability, access to parks and playgrounds, and neighborhood safety [8]–[13].

In the domain of the contextual *social* environment, recent studies in the US and Europe have demonstrated an association between individual and/or neighborhood-level *social capital* and physical activity [4], [6], [9], [14]–[16]. Social capital has been broadly defined as features of social organization that facilitate cooperation for the achievement of common goals. Several mechanisms have been hypothesized as to why community social capital could promote physical activity, including: a) *informal social control* which contributes to the prevention of juvenile delinquency, thereby promoting perceptions of safety among residents, and thus encouraging them to exercise outdoors; b) *collective efficacy* among residents to improve access to resources for physical activity (e.g. building bike paths, or maintaining the upkeep of public spaces and parks); and c) the diffusion of healthy norms (e.g. seeing the neighbors go out to jog every day) as well as social influence (e.g. group calisthenics or *tai chi* by senior citizens commonly encountered in China, as well as Japanese croquet practiced in Japan).

Indeed, the strength of social capital in U.S. communities has been linked to higher levels of physical activity among U.S. residents [9], [15], [16], although a null study has also been reported in Europe [6]. Studies using individual-level perceptions of social capital have also consistently reported an inverse relationship with physical inactivity [4], [6], [9], [14].

The operational definition and measurement of social capital remains somewhat contested. However, an emerging consensus has defined two axes of measurement, including: a) the distinction between cognitive dimensions (e.g. perceived trust of others in the community) versus structural/behavioral dimensions (e.g. participation in civic and voluntary groups), and b) the distinction between bonding social capital and bridging social capital [17], [18].

Bonding social capital refers to connections between members of a community who are similar in terms of their social identity, whether defined by social class, race/ethnicity, or other characteristics [17], [19]. Bridging social capital refers to connections between members of a community who are unlike each other, but are more or less equal in terms of their status and power [17], [19]. Previous studies have examined the link between social capital and physical activity in only one or the other of these dimensions. For example, some studies have only looked at structural social capital [4], [6], while others have measured both cognitive and structural social capital, but failed to distinguish between bonding and bridging capital [9], [16].

Our aim in this study was to examine whether individual-level social capital was associated with physical inactivity in a population-based sample in Japan. We defined and measured social capital in all four domains – i.e. cognitive, structural, bonding or bridging.

## Methods

### Ethics Statement

The survey was conducted by the City Government of Okayama. At the stage of releasing the data to researchers, personal identifiers (names, full addresses) were stripped from the dataset. The study was reviewed and approved by the Harvard School of Public Health Institutional Review Board.

### Participants

The Okayama Social Capital Study was a population-based cross-sectional study with the primary purpose of investigating the associations between social capital and health behavior within Okayama city, a mid-sized urban city in western Japan with a population of about 700,000 people. The survey was administered by the City Government of Okayama. A cluster sampling approach was used to randomly select 4,000 residents living in 87 school districts in Okayama city. Within each school district, 200 residents were randomly chosen using the Basic Resident Register (an enumeration of every residence in Japan managed by the national government and prefecture) as the sampling frame. In total, 4000 people, aged 20–80 years, were selected and mailed a questionnaire in February 2009. Sixty one questionnaires were returned because of unknown address, death, or other reasons, and of the remaining 3939 questionnaires, 2260 were completed and returned (response rate: 57.4%).

### Measures

Community social capital was assessed in both the cognitive and structural domains. Cognitive social capital was assessed by a single item inquiring about the respondent's perception of trust in their community: “People in my neighborhood trust each other.” The responses were selected from a Likert scale, ranging from “strongly disagree” (6.6%), “somewhat disagree” (7.4%), “undecided” (29.0%), “somewhat agree” (38.6%), and “strongly agree” (17.2%). “Strongly disagree”, “somewhat disagree”, and “undecided” were clumped into low trust (43.0%), “somewhat agree” was assigned to mid trust (38.6%), and “strongly agree” was assigned to high trust (17.3%). The structural/behavioral dimension of social capital was assessed through a single question: “Do you participate in community activities such as neighborhood associations, block meetings, and women's associations?” In addition, subjects who answered as participating in such activities were asked whether other participants in the groups were “similar to them” or “different from them” regarding sex, age, and occupation. When the participant reported that other participants were similar to them, the group was classified as belonging to the “bonding” type, and when different from them, as “bridging” type. In other words, we defined bonding and bridging social capital based on the participants' perception, rather than the actual distribution of sex, age, and occupation of the members taking part in community activities.

Physical activity was assessed with a single question: “How often do you participate in sports or physical exercise?” The Likert scale responses were “almost never”, “1–3 days a month”, “1–2 days a week”, “almost every day”. Since the concern of the study was physical inactivity, the options were combined into two categories as “active” and “inactive”. Specifically, “almost never” was designated as “inactive” and all other responses (“1–3 days a month”, “1–2 days a week”, and “almost everyday”) were designated as “active”.

We considered the following variables as potential confounders: sex, age (continuous variable), body mass index (BMI) calculated from height and weight (continuous variable), years of education (under 12 years, 13–15 years, 16 years or more), family structure living together (one generation, two generations, others), self-rated health (good, very good, excellent designated as good; fair, poor designated as poor), and mental status (depressed, not depressed). Mental status was assessed as the number of days feeling depressed in the past month, designating none to several days as “not depressed”, and others to “depressed”.

### Statistical analyses

We performed sequential logistic regressions to examine the relationships between social capital (cognitive and structural) and physical inactivity. The three response categories in each of the social capital variables (trust and social participation) were recoded into dummy variables, and analyzed independently in separate regression models. In each model, low trust and lack of social participation were set as the referent category. In model 1, we regressed physical inactivity on each aspect of social capital, adjusting for age and sex. In model 2, we additionally controlled for educational attainment and family structure. In model 3, we additionally controlled for self-rated health, mental status, and BMI. Finally, using model 3, we imputed missing data using the multiple imputation method [20] and created 10 complete datasets of the 2260 respondents, analyzed each dataset and pooled the results. We calculated the odds ratios (ORs) and 95% confidence intervals (CIs) for physical inactivity associated with each domain of social capital.

*P*-values for trend were only calculated within cognitive social capital, treating the three categories as ordinal variables. A *P*-value of less than 0.05 (two-sided test) was considered statistically significant.

All analyses were performed using STATA/SE 10.1 (StataCorp, College Station, TX, USA).

## Results

The demographic and health characteristics of all the subjects (*n* = 2260) in the study are shown in Table 1. Among total participants, 69% were physically active and 29% were physically inactive. The average age of all participants was 52.9 years, and the average BMI was 22.4 kg/m^2^, which is close to the ideal BMI. Among total participants, 43% reported low trust, and 36% of the participants were involved in community activities (either bonding or bridging).

**Table 1:** Characteristics of study subjects in Okayama, Japan (2009).

	Total
**Mean age, years (SD)**	52.9 (16.6)
**Mean BMI, kg/m^2^ (SD)**	22.4 (3.3)
**Sex, n (%)**	
Men	886 (39.2)
Women	1301 (57.6)
Missing	73 (3.2)
**Years of education, n (%)**	
<12 years	1095 (48.5)
13–15 years	462 (20.4)
≥16 years	578 (25.6)
Missing	125 (5.5)
**Family structure, n (%)**	
One generation	781 (34.6)
Two generations	1046 (46.3)
Others	352 (15.6)
Missing	81 (3.6)
**Self-rated health, n (%)**	
Good	1799 (79.6)
Poor	392 (17.4)
Missing	69 (3.1)
**Mental status, n (%)**	
Not depressed	1690 (74.8)
Depressed	498 (22.0)
Missing	72 (3.2)
**Trust of others in neighborhood, n (%)**	
Low	971 (43.0)
Mid	872 (38.6)
High	390 (17.3)
Missing	27 (1.2)
**Social participation, n (%)**	
None	1249 (55.3)
Bridging	612 (27.1)
Bonding	202 (8.9)
Missing	197 (8.7)
**Physical activity, n (%)**	
Active	1556 (68.8)
Inactive	654 (28.9)
Missing	50 (2.2)

BMI =  body mass index; SD =  standard deviation.

Table 2 shows the distribution of subjects according to each dimension of social capital. Subjects with low trust tended to report low bonding social capital, while subjects with high social capital tended to exhibit high bridging social capital. The *P* value for Pearson's chi-square test between trust and social participation was *P*<0.001.

**Table 2:** Number of subjects by cognitive and structural social capital status in Okayama, Japan (2009).

	Social participation			
Trust	None	Bridging	Bonding	Total
Low	660	186	69	915
Mid	445	269	84	798
High	133	149	49	331
Total	1238	604	202	2044

Table 3 shows the distribution of demographic and health characteristics of the study sample according to type and level of social capital. Subjects with higher levels of trust tended to be older, report fewer years of education, more likely to be living in a smaller family, and were less depressed. Those reporting lower levels of social participation tended to be younger, more educated, and also more likely to be depressed. Compared with those with high bridging social capital, participants with bonding social capital tended to be older, report fewer years of education, and were more likely to be depressed.

**Table 3:** Characteristics of study subjects according to social capital status in Okayama, Japan (2009).

	Trust			Social participation		
	Low	Mid	High	None	Bridging	Bonding
**Number of subjects**	(n = 971)	(n = 872)	(n = 390)	(n = 1249)	(n = 612)	(n = 202)
**Mean age, years (SD)**	48.5 (16.0)	53.4 (15.9)	62.2 (13.9)	48.0 (16.8)	57.1 (12.9)	59.1 (14.0)
**Mean BMI, kg/m^2^ (SD)**	22.0 (3.4)	22.5 (3.3)	22.8 (3.1)	22.2 (3.4)	22.5 (3.2)	22.6 (3.2)
**Sex, n (%)**						
Men	381 (39.2)	345 (39.6)	157 (40.3)	505 (40.4)	237 (38.7)	77 (38.1)
Women	558 (57.5)	502 (57.6)	222 (56.9)	708 (56.7)	361 (59.0)	120 (59.4)
Missing	32 (3.3)	25 (2.9)	11 (2.8)	36 (2.9)	14 (2.3)	5 (2.5)
**Years of education, n (%)**						
<12 years	434 (44.7)	399 (45.8)	248 (63.6)	555 (44.4)	301 (49.2)	110 (54.5)
13–15 years	224 (23.1)	181 (20.8)	54 (13.9)	270 (21.6)	131 (21.4)	43 (21.3)
≥16 years	257 (26.5)	253 (29.0)	64 (16.4)	364 (29.1)	155 (25.3)	40 (19.8)
Missing	56 (5.8)	39 (4.5)	24 (6.2)	60 (4.8)	25 (4.1)	9 (4.5)
**Family structure, n (%)**						
One generation	328 (33.8)	281 (32.2)	168 (43.1)	434 (34.8)	201 (32.8)	69 (34.2)
Two generations	464 (47.8)	420 (48.2)	152 (39.0)	599 (48.0)	291 (47.6)	84 (41.6)
Others	142 (14.6)	148 (17.0)	55 (14.1)	178 (14.3)	103 (16.8)	43 (21.3)
Missing	37 (3.8)	23 (2.6)	15 (3.9)	38 (3.0)	17 (2.8)	6 (3.0)
**Self-rated health, n (%)**						
Good	758 (78.1)	720 (82.6)	304 (78.0)	990 (79.3)	518 (84.6)	162 (80.2)
Poor	183 (18.9)	129 (14.8)	74 (19.0)	227 (18.2)	80 (13.1)	32 (15.8)
Missing	30 (3.1)	23 (2.6)	12 (3.1)	32 (2.6)	14 (2.3)	8 (4.0)
**Mental status, n (%)**						
Not depressed	673 (69.3)	695 (79.7)	306 (78.5)	909 (72.8)	496 (81.1)	157 (77.7)
Depressed	268 (27.6)	153 (17.6)	70 (18.0)	308 (24.7)	103 (16.8)	38 (18.8)
Missing	30 (3.1)	24 (2.8)	14 (3.6)	32 (2.6)	13 (2.1)	7 (3.5)
**Physical activity, n (%)**						
Physically active	611 (62.9)	628 (72.0)	304 (78.0)	809 (64.8)	452 (73.9)	151 (74.8)
Physically inactive	346 (35.6)	230 (26.4)	69 (17.7)	425 (34.0)	149 (24.4)	42 (20.8)
Missing	14 (1.4)	14 (1.6)	17 (4.4)	15 (1.2)	11 (1.8)	9 (4.5)

BMI =  body mass index; SD  =  standard deviation.

Table 4 shows the association between cognitive and structural social capital and physical inactivity. Across all models, high trust was associated with lower odds of physical inactivity. High trust was associated with a 42% lower odds of physical inactivity (odds ratio (OR) = 0.58, 95% confidence interval (CI) = 0.42–0.79), compared with participants with low trust after controlling for covariates (multiple imputation). The *P*-values for trend across levels of trust were also statistically significant (*P*<0.005 for all models), suggesting a dose-response effect. Both bridging and bonding social capital were about equally (and statistically significantly) associated with lower odds of physical inactivity in the crude model (bridging, OR = 0.63, 95% CI = 0.50–0.78; bonding, OR = 0.53, 95% CI = 0.37–0.76). However, after adjusting for covariates and imputing data, the associations became only marginally significant (bridging, OR = 0.79, 95% CI = 0.62–1.00; bonding, OR = 0.71, 95% CI = 0.48–1.03) (multiple imputation).

**Table 4:** Odds ratios for physical inactivity associated with social capital within neighborhoods.

	Crude OR (95% CI)	Adjusted OR (95% CI)			
		Model 1^a^	Model 2^b^	Model 3^c^	Multiple imputation^c^
**Cognitive Social Capital**					
Low	1.00	1.00	1.00	1.00	1.00
Mid	0.65 (0.53–0.79)	0.73 (0.59–0.91)	0.73 (0.59–0.91)	0.78 (0.62–0.97)	0.73 (0.59–0.90)
High	0.40 (0.30–0.54)	0.57 (0.42–0.78)	0.59 (0.43–0.81)	0.64 (0.46–0.88)	0.58 (0.42–0.79)
p for trend	<0.001	<0.001	<0.001	0.002	<0.001
**Structural Social Capital**					
None	1.00	1.00	1.00	1.00	1.00
Bridging	0.63 (0.50–0.78)	0.80 (0.64–1.02)	0.78 (0.61–0.99)	0.81 (0.63–1.04)	0.79 (0.62–1.00)
Bonding	0.53 (0.37–0.76)	0.71 (0.48–1.04)	0.70 (0.48–1.04)	0.73 (0.50–1.09)	0.71 (0.48–1.03)

OR =  odds ratio; CI =  confidence interval.

a Simultaneously adjusted for sex, age.

b Simultaneously adjusted for sex, age, educational attainment, family structure.

c Simultaneously adjusted for sex, age, educational attainment, family structure, self-rated health, mental status, BMI.

## Discussion

This study suggests that higher levels of cognitive social capital (trust of neighbors) is associated with lower odds of physical inactivity, even after controlling for age, sex, years of education, family structure, self-rated health, mental status, and BMI. On the other hand, although social participation (structural social capital) was associated with lower odds of physical inactivity, the 95% confidence intervals included the null value after statistical control for potential confounding variables. Moreover, the associations of group participation with physical inactivity were roughly similar for groups that were of the bonding and bridging variety.

To our knowledge, this is the first study to investigate the association between individual-level social capital and physical activity in the Japanese population. In a U.S. sample, Greiner et al. (2004) showed that physical activity was more strongly associated with structural social capital compared with cognitive social capital [9]. Our Japanese sample exhibited the opposite trend, i.e. stronger association between trust and physical activity compared to social participation, One explanation of this result may be that Japanese people participating in social activities tend to have more social obligations and as a result do not have enough time to devote to physical activity. Alternatively, our finding could have been influenced by measurement error. Although trust was categorized into three levels, social participation was only measured by a dichotomous response (yes/no). Furthermore, the relatively low response rate of 57% may have induced selection bias and resulted in a distorted conclusion. Since information about nonrespondents were unavailable, we should be careful in the interpretation of the results. A limitation of our study is that we did not ask about potential pathways and mechanisms that could explain the association between social capital and physical activity. For example, generalized trust of neighbors could affect physical activity through feelings of security in the neighborhood connected with trust, which encourage residents to practice physical activity [13], [21]. However, the survey did not inquire specifically about perceptions of safety.

A second limitation of our study is that we confined our analysis to examining individual-level perceptions and individual behaviors (social participation). To the extent that social capital has been conceptualized as a *contextual* influence on behavior, the ideal analytical strategy would have been to conduct a multi-level study, whereby social capital perceptions *aggregated to the level of the neighborhood* is linked to individual-level behaviors.

A third limitation of our study is that it was cross-sectional, and hence, we could not exclude the possibility of reverse causation. Reverse causation is of particular concern for behavioral measures of social capital (i.e. social participation), because by definition, individuals who are physically active are more likely to participate in community organizations. However, our findings indicated that the cognitive dimension of social capital (trust) was more robustly associated with physical activity than the behavioral dimension. It is (somewhat) harder to argue that being physically active causes people to trust others in their community.

Arguably the most significant limitation of our study is the fact that we used a single-question self-report questionnaire to assess the information of cognitive and structural domains of social capital from the participants, making data susceptible to measurement error and misclassification. The questionnaire has not been subjected to validity or reliability testing in this study, although similar items for cognitive social capital have been employed in past studies [22], [23]. The use of single questions in obtaining information may mitigate our ability to further analyze the specific domains of cognitive social capital. The question for structural social capital was determined through definitions proposed by Szreter and Harpham [17], [18], since very few previous studies have distinguished between bonding and bridging social capital. A further concern is that we did not inquire about co-morbid conditions and diagnoses. Disease and physical inactivity may be strongly related, but instead, we controlled for this using self-rated health as a proxy for physical health status.

We have used the term “physical inactivity” throughout the text to refer to the lack of physical activity. We must acknowledge, however, that physical inactivity is regarded as a state in which body movement is minimal [24], often measured as modifiable or necessary sedentary activities [25], and does not exactly refer to the lack of physical activity. In other words, it is possible for an individual to be simultaneously physically active (e.g. exercising on the treadmill for an hour every day) *and* physically inactive (spending the remaining waking hours working in front of a computer screen). Epidemiological studies have established that both physical inactivity (or sedentarism) and the lack of physical activity are risk factors for chronic disease [3], [26]. Since our survey did not specifically inquire about sedentarism, our health outcome should be interpreted as the *lack* of physical activity.

In conclusion, our study suggests a protective association of individual-level social capital, especially trust, on physical activity among Japanese adults. Although social participation may also have a protective effect, the association was weaker, and we did not find a difference in the association comparing bonding and bridging structural social capital. In further studies, it would be desirable to assess information on neighborhood-level environmental and social capital employing a multi-level analytical framework. To overcome reverse causation, it would also be desirable to collect longitudinal information from participants.
